# Proenkephalin as a biomarker correlates with acute kidney injury: a systematic review with meta-analysis and trial sequential analysis

**DOI:** 10.1186/s13054-023-04747-5

**Published:** 2023-12-07

**Authors:** Li-Chun Lin, Min-Hsiang Chuan, Jung-Hua Liu, Hung-Wei Liao, Leong L. Ng, Martin Magnusson, Amra Jujic, Heng-Chih Pan, Vin-Cent Wu, Lui G. Forni

**Affiliations:** 1 Department of Internal Medicine, National Taiwan University Hospital, Taipei, Taiwan; 2 Department of Internal Medicine, Chi Mei Medical Center, Tainan, Taiwan; 3 Department of Communication, National Chung Cheng University, Chiayi, Taiwan; 4 Division of Nephrology, Department of Internal Medicine, Wan Fang Hospital, Taipei Medical University, Taipei, Taiwan; 5 Division of Nephrology, Department of Internal Medicine, School of Medicine, College of Medicine, Taipei Medical University, Taipei, Taiwan; 6 Department of Cardiovascular Sciences, University of Leicester, Glenfield Hospital, Groby Road, Leicester, UK; 7 National Institute for Health Research Leicester Biomedical Research Centre, Glenfield Hospital, Groby Road, Leicester, UK; 8 Department of Clinical Sciences, Lund University, Malmö, Sweden; 9 Department of Cardiology, Skåne University Hospital, Malmö, Sweden; 10 Wallenberg Center for Molecular Medicine, Lund University, Lund, Sweden; 11 Hypertension in Africa Research Team (HART), North-West University, Potchefstroom, South Africa; 12 Division of Nephrology, Department of Internal Medicine, Keelung Chang Gung Memorial Hospital, 222 Mai-Jin Road, Keelung, 204 Taiwan; 13 College of Medicine, Chang Gung University, Taoyuan, Taiwan; 14 Community Medicine Research Center, Keelung Chang Gung Memorial Hospital, Keelung, Taiwan; 15 Graduate Institute of Clinical Medicine, College of Medicine, National Taiwan University, Taipei, Taiwan; 16 Division of Nephrology, Department of Internal Medicine, National Taiwan University Hospital, Taipei, Taiwan; 17 NSARF (National Taiwan University Hospital Study Group of ARF), TAIPAI, (Taiwan Primary Aldosteronism Investigators), and CAKS (Taiwan Consortium for Acute Kidney Injury and Renal Diseases), Taipei, Taiwan; 18 Department of Critical Care, Royal Surrey Hospital Foundation Trust, Guildford, UK; 19 Department of Clinical and Experimental Medicine, Faculty of Health Sciences, University of Surrey, Guildford, UK

**Keywords:** Proenkephalin, Acute kidney injury, Biomarker, Meta-analysis

## Abstract

**Background:**

Proenkephalin A 119-159 (PENK) is freely filtered in the glomerulus with plasma levels correlating with glomerular filtration rate. Therefore, PENK has been proposed as an early indicator of acute kidney injury (AKI) although its performance is dependent on the clinical setting. This meta-analysis aimed to investigate the correlation between PENK levels and the development of AKI.

**Methods:**

We conducted a comprehensive search on the PubMed, Embase, Cochrane databases, the website ClinicalTrials.gov and Cnki.net until June 26, 2023. Summary receiver operating characteristic (SROC) curves were used to amalgamate the overall test performance. Diagnostic odds ratio (DOR) was employed to compare the diagnostic accuracy of PENK with other biomarkers. Quality of the evidence was assessed using the Grading of Recommendations, Assessment, Development and Evaluations (GRADE) criteria.

**Results:**

We incorporated 11 observational studies with 3969 patients with an incidence of AKI of 23.4% (929 out of 3969 patients) with the best optimal cutoff value of PENK for early detection of AKI being 57.3 pmol/L. The overall sensitivity and specificity of PENK in identifying AKI were 0.69 (95% CI 0.62–0.75) and 0.76 (95% CI 0.68–0.82), respectively. The combined positive likelihood ratio (LR) stood at 2.83 (95% CI 2.06–3.88), and the negative LR was 0.41 (95% CI 0.33–0.52). The SROC curve showcased pooled diagnostic accuracy of 0.77 (95% CI 0.73–0.81). Interestingly, patients with a history of hypertension or heart failure demonstrated a lower specificity of PENK in correlating the development of AKI.

**Conclusion:**

Our results indicate that PENK possesses significant potential as a biomarker for the early detection of the development of AKI, using a cutoff point of 57.3 pmol/L for PENK.

**Supplementary Information:**

The online version contains supplementary material available at 10.1186/s13054-023-04747-5.

## Background

Acute kidney injury (AKI) is common and its development is associated with increased mortality and morbidity including an increased likelihood of developing chronic kidney disease (CKD) [1]. The importance of detecting AKI cannot be overstated since early detection dictates the timing of therapeutic measures and informed decisions in clinical settings [2–5]. Conventional indicators like serum creatinine (SCr) demonstrate a delayed response following the initial injury, and they are also influenced by a multitude of variables (e.g., body composition) [6]. Furthermore, the effectiveness of novel biomarkers in predicting AKI can vary depending on the clinical circumstances, reflecting the diverse etiologies responsible for AKI [7, 8]. Nonetheless, the use of such biomarkers will equip healthcare professionals with a more in-depth, real-time comprehension of kidney health, potentially leading to improved patient outcomes [9, 10]. Proenkephalin A 119-159 (PENK) is a persistent precursor fragment of the transient enkephalin product and has emerged as a promising and innovative biomarker for AKI [11]. Enkephalins, which are endogenous opioids, activate µ- and δ-opioid receptors of which the highest density outside the central nervous system is found in the kidney [12]. While their exact function is unclear, it appears that they play a possible regulatory role with a strong inverse relationship observed between plasma PENK concentration and measured glomerular filtration rate determined by iothalamate clearance in individuals with normal renal function [13]. PENK is stable after collection, not affected by sex, age or protein binding, and has a long in vivo half-life. Because it is solely filtered by the glomerulus, this renders it an excellent candidate biomarker for the early detection of AKI [14].

PENK has been studied as an early indicator of AKI across diverse clinical cohorts, but the results reported show variable performance. In patients with sepsis, PENK appears to be a dependable early indicator of AKI, whereas in patients with CKD developing AKI post-exposure to contrast medium, no differences in the baseline PENK levels between the AKI and non-AKI cohorts were observed [15, 16]. Due to the diverse outcomes observed, we undertook an exhaustive systematic review together with meta-analysis and trial sequential analyses, to investigate the potential of plasma PENK as a marker of AKI.

## Methods

### Data sources and search strategy

Two reviewers (LC Lin and HW Liao) independently searched the Cochrane Central Register of Controlled Trials (CENTRAL), PubMed, EMBASE, MEDLINE, ClinicalTrials.gov and Cnki.net until Jun 26, 2023, using terms associated with AKI (“acute renal failure,” “acute kidney impairment,” “acute kidney insufficiency” and “AKI”) and PENK (“proenkephalin A,” “proenkephalin A 119-159,” “PenKid” and “PENK”). The search strategies are listed in the Additional file 1. We also manually checked the reference list of related review articles, editorials and identified studies to identify any further randomized controlled trials (RCTs). The full texts of potentially eligible RCTs and observational studies were retrieved and evaluated for inclusion. Additionally, we contacted the original authors to acquire additional information in cases where the data were incomplete.

This systematic review and meta-analysis was performed in accordance with the Preferred Reporting Items of Systematic Reviews and Meta-Analyses (PRISMA) recommendation and Cochrane methods. The study protocol was registered in PROSPERO [CRD42023424693].

### Inclusion and exclusion criteria

Studies meeting the inclusion criteria were identified based on the following criteria: (1) evaluation of the diagnostic performance of PENK for AKI in adult patients and (2) provision of comprehensive information, including sample size, sensitivity and specificity at a designated cutoff value, thereby facilitating the pooling of data for accuracy analysis. Exclusion criteria encompassed duplicate publications, case reports, conference abstracts and non-original articles, such as reviews and commentaries. Language restrictions were not imposed.

### Study selection and data extraction

Two reviewers (LC Lin and HW Liao) independently reviewed full-text articles, individual study protocols and the template for case report forms and evaluated the risk of bias in methodology. Discrepancies were resolved through discussions with a third investigator (VC Wu). The data extracted from the enrolled studies included the first author, publication year, study designs, sample/event sizes, clinical settings, patients' characteristics (age, sex, comorbidities and baseline renal function), timing of PENK measurement, AKI criteria, study endpoint and diagnostic accuracy assessment (specifically the cutoff value of PENK for the early diagnosis of AKI along with the corresponding sensitivity and specificity). The baseline characteristics of included studies are illustrated in Table 1.

**Table 1:** Characteristics of included studies

Study (year)	Population setting	Total patient	AKI (%)	Mean age (y)	Male (%)	HTN (%)	DM (%)	CKD (%)	CHF (%)	Baseline SCr (mg/dL)^a^	Endpoint	Follow-up duration	AKI criteria	Timing of PENK measurement	PENK assay
Shah et al. [29]	Cardiac surgery	92	20 (21.7)	66	98.8	35.9	58.7	13	N/A	1.26 and 0.99	Post-op AKI	Until discharge	AKIN	Pre-op	ILMA
Mossanen et al. [30]	Cardiac surgery	107	21 (19.6)	69	72	75.7	36.4	36.4	9	1.0 and 0.9	Post-op AKI	4 days post-op	KDIGO	Pre-op	ILMA
Kim et al. [31]	Sepsis	167	41 (24.6)	70	59.3	N/A	N/A	N/A	N/A	N/A	AKI	2 days post enrollment	KDIGO	Upon enrollment	ILMA
Ng et al. [32]	Acute heart failure	1572	236 (15)	76	63.1	71.0	32.4	34.5	44.2	1.61 and 1.39	AKI	5 days post admission	KDIGO	Admission	ILMA
Hollinger et al. [33]	Sepsis	582	360 (61.9)	66	62.4	50.5	27.5	13.1	N/A	1.35	Persistent AKI by day 7	7 days post admission	KDIGO	Admission	ILMA
Breidthardt et al. [16]	CKD with contrast medium exposure	111	7 (6.3)	77	62.2	84.7	43.2	100	33	1.60 and 1.50	Contrast-induced AKI	2 days post contrast medium exposure	SCr ≥ 25% or 0.5 mg/dL ↑ from baseline within the first 48 h after contrast medium exposure	Day 0	ILMA
Rosenqvist et al. [15]	Sepsis	588	94 (16)	73	51.0	N/A	19.4	7.7	18.9	1.75 and 0.90	AKI	7 days post presentation	AKIN stage 3 or SCr ≥ 50% ↑ from baseline with an initial SCr > 2 mg/dL within 7 days	Emergency department presentation	ILMA
Molvin et al. [34]	Acute heart failure	530	67 (12.6)	76	60.2	96.8	36.6	12.6	53.8	1.37	AKI	2 days post admission	SCr ≥ 50% or 0.3 mg/dL ↑ from baseline within 48 h after admission	Admission	ILMA
Liu et al. [35]	Sepsis	42	16 (38.1)	N/A	N/A	N/A	N/A	N/A	N/A	N/A	AKI	N/A	KDIGO	Within 48 h after the diagnosis of sepsis	N/A
Lima et al. [36]	Liver transplant	57	36 (63.2)	58	61.4	N/A	N/A	N/A	N/A	1.00 and 0.82	Post-op AKI stage 2–3	7 days post-op	KDIGO	Pre-op	ILMA
Zhao et al. [37]	Acute heart failure	121	31 (25.6)	66	59.5	64.5	26.4	13.2	N/A	1.95 and 0.88	AKI	Until discharge	KDIGO	Admission	ELISA

AKI, Acute kidney injury; AKIN, Acute Kidney Injury Network; CHF, congestive heart failure; CKD, chronic kidney disease; DM, diabetes mellitus; ELISA, enzyme-linked immunosorbent assay; HTN, hypertension; ILMA, immunoluminometric assay; KDIGO, Kidney Disease: Improving Global Outcomes, N/A, not applicable; PENK, proenkephalin A 119-159; Pre-op, preoperative; Post-op, postoperative; SCr. serum creatinine

^a^Values given separately for patients with and without AKI in some studies

### Outcome

The primary outcome of this study was the development of AKI, treated as a binary outcome.

### Quality assessment

The risk of bias and applicability of the individual study were evaluated using the Quality Assessment of Diagnostic Accuracy Studies-2 (QUADAS-2) tool [17, 18]. Four crucial domains were assessed, namely patient selection, index test, reference standard and flow and timing. Each domain was categorized as having a low, unclear or high risk of bias. Any disagreements in the quality assessment were resolved by discussion and consensus [19]. The findings of the assessment were then visually depicted in a summarized graphical format.

### Pre-specified subgroup analysis

We hypothesized that multiple factors, including baseline characteristics such as age, sex and pre-existing conditions such as hypertension, diabetes, CKD and cardiac events, could have a substantial impact on the observed patient outcomes in the reported studies. We also considered the use of mean values for grouping, and whether the studies included surgical patients exclusively or a combination of surgical and medical patients, along with patients suffering from sepsis. The AKI criteria employed—RIFLE (Risk, Injury, Failure, Loss, end-stage renal disease (ESRD)), AKIN (Acute Kidney Injury Network), KDIGO (Kidney Disease: Improving Global Outcomes), and the severity of AKI and the variation in follow-up durations (greater than 2 days or less than or equal to 2 days) were also examined. Additionally, to evaluate the potential influence of small-study effects on overestimation, we stratified our analysis based on study size [20].

### Data synthesis and statistical analysis

The determination of true positives, true negatives, false positives and false negatives was carried out by utilizing the sample size, event rate, sensitivity and specificity information obtained from each respective study. In instances where the sensitivity and specificity values were not explicitly provided in the studies, we employed WebPlotDigitizer (version 4.6) to digitally extract the data from the receiver operating characteristic (ROC) curve [21]. The overall diagnostic performance of PENK was evaluated by utilizing a summary receiver operating characteristic (SROC) curve along with measures such as pooled sensitivity, specificity, positive likelihood ratio (LR) and negative LR [22]. The optimal cutoff point for PENK in association with the development of AKI was ascertained through the methodology introduced by Steinhauser et al. [23]. We adopted the logistic distribution assumption and employed the model that minimized the restricted maximum likelihood (REML). A weighting parameter of 0.5 was applied to ensure an equal emphasis on sensitivity and specificity. The optimal cutoff value was determined as the point that maximizes the Youden index [24]. When examining the diagnostic accuracy of AKI using PENK, we utilized the diagnostic odds ratio (DOR) for comparison. We compared the diagnostic capability of PENK with neutrophil gelatinase-associated lipocalin (NGAL), a kidney tubular damage marker known for its good diagnostic performance [10].

Fagan diagrams were used to examine the clinical applicability of PENK as an early indicator of AKI. Heterogeneity was quantified using the *I*^2^ statistics, with substantial heterogeneity defined as *I*^2^ > 50%. Subgroup analysis and meta-regression were conducted to investigate potential sources of heterogeneity observed between the included studies. Funnel plots were utilized to assess the presence of publication bias. Moreover, to account for type-I and type-II errors and predetermined number of patients was reached, trial sequential analysis (TSA) was performed. The TSA was set with a power level of 90% and a two-tailed α level of 0.05 [25–28]. All statistical analyses were conducted using Stata software (version 16) with the midas package, R software (version 3.6.0) or TSA software (version 0.9.5.10 Beta).

## Results

### Search results and study characteristics

A summary of the study selection process is provided in Additional file 1. The initial database search yielded 175 articles. Following the removal of duplicates, the titles and abstracts of 81 articles were assessed. Eventually, a total of 23 studies met the eligibility criteria for a full-text review. Among these, 11 observational studies comprising 3969 patients reported data on the occurrence of AKI with PENK and were included in the meta-analysis [15, 16, 29–37]. We contacted the corresponding authors of nine studies via email for missing data clarification, and two provided additional information [32, 34]. The population characteristics and performance of plasma PENK in each individual study are summarized in Tables 1 and 2. The mean baseline SCr levels ranged from 0.88 to 1.95 mg/dL (77–173 µmol/l). The included studies encompassed a broad range of clinical settings, including sepsis (three studies, 1379 patients), acute heart failure (three studies, 2223 patients), cardiac surgery (two studies, 199 patients), liver transplant (one study, 57 patients) and contrast medium exposure (one study, 111 patients). Among the included studies, seven studies employed the KDIGO criteria for defining AKI, while two studies utilized the AKIN criteria. Additionally, two studies specifically focused on advanced stages of AKI, while the other studies encompassed any stage of AKI. The duration of follow-up varied across the studies.

**Table 2:** Performance characteristics of plasma PENK in individual studies

Study (year)	No. of patients				PENK cutoff (pmol/L)	Sensitivity	Specificity	AUC (95% CI)
	True-positive	False-positive	False-negative	True-negative				
Shah et al. [29]	11	9	10	62	N/A	0.55	0.86	0.683 (N/A)
Mossanen et al. [30]	12	9	30	56	93.2	0.59	0.65	0.651 (N/A)
Kim et al. [31]	27	14	26	100	154.5	0.66	0.79	0.725 (0.651–0.791)
Ng et al. [32]	132	104	454	882	116.7	0.56	0.66	0.642 (0.605–0.680)
Hollinger et al. [33]	256	104	34	188	84.2	0.71	0.85	0.854 (0.823–0.884)
Breidthardt et al. [16]	5	2	32	72	N/A	0.71	0.69	0.60 (0.34–0.86)
Rosenqvist et al. [15]	67	27	124	370	N/A	0.71	0.75	0.758 (0.702–0.815)
Molvin et al. [34]	43	24	190	273	104	0.64	0.59	0.652 (0.583–0.721)
Liu et al. [35]	10	6	0	26	67.0	0.61	1.00	0.884 (0.738–0.965)
Lima et al. [36]	31	5	10	11	55.3	0.86	0.52	0.69 (0.54–0.83)
Zhao et al. [37]	28	3	16	74	57.0	0.90	0.82	0.808 (0.54–0.83)

AUC, Area under curve; CI, confidence interval; N/A, not applicable; PENK, proenkephalin A 119-159

### Quality of the enrolled trials

The comprehensive evaluation of study quality was guided by the QUADAS-2 framework (Additional file 1: Fig. S1). Within the patient selection domain, it was determined that one study incurred a high risk of bias, attributed to the non-enrollment of consecutive patients. Furthermore, two studies were classified as having an unclear risk of bias due to insufficient information regarding patient selection. However, all studies under scrutiny demonstrated a low risk of bias in both the index test and reference standard domains. Regarding flow and timing, three studies warranted an unclear risk of bias as not all participants were included in the analysis [17, 18].

**Fig. 1:**
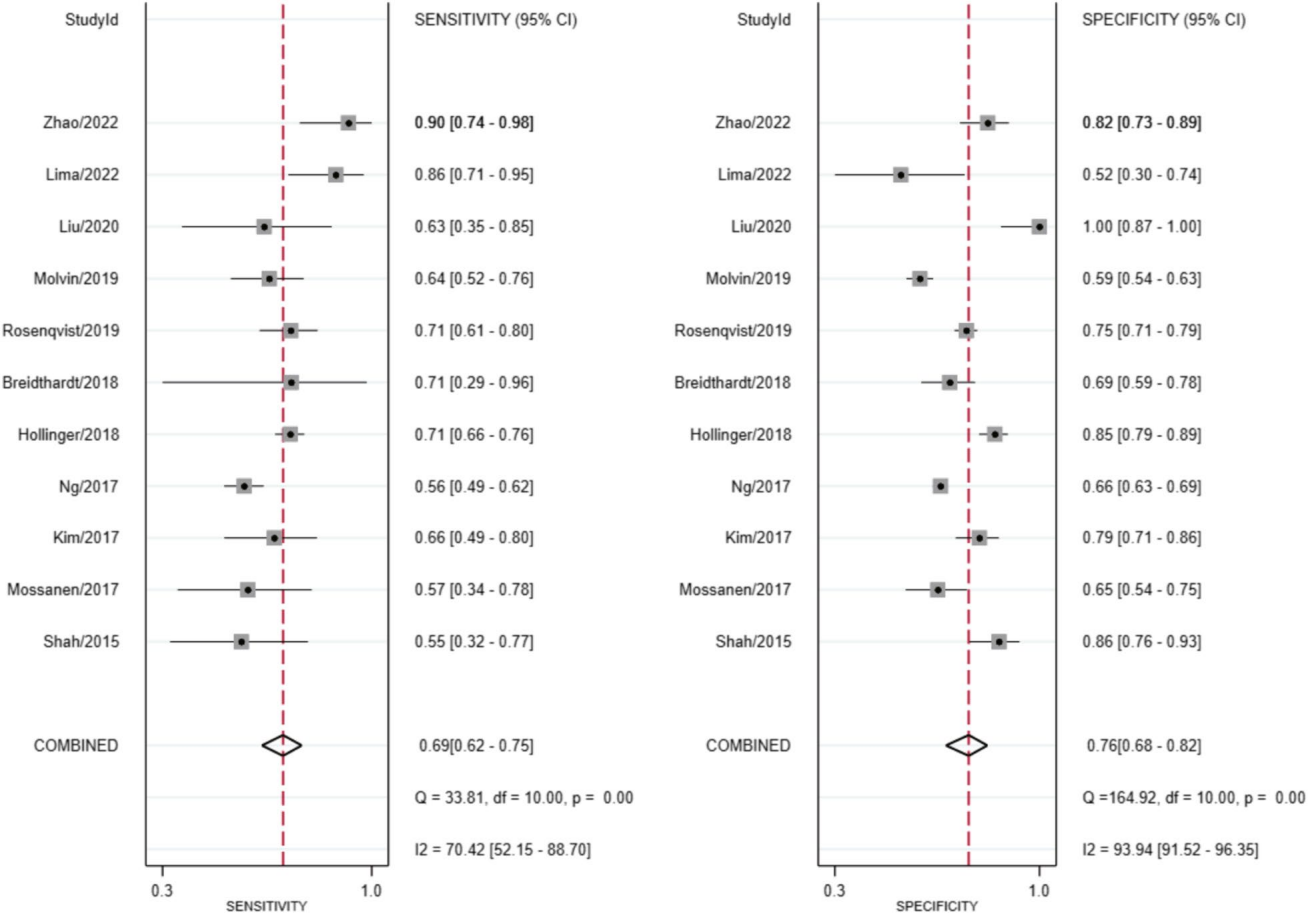
Forest plot of the pooled sensitivity and specificity of PENK for the early detection of AKI in all studies. AKI, Acute kidney injury; PENK, proenkephalin A 119-159

### Primary outcome

The incidence of AKI was determined from the complete set of included studies giving a total patient pool of 3,969 individuals. Of these, 929 developed AKI (23.4%). The diagnostic values, threshold levels and the sensitivity and specificity of PENK from each study are shown (Table 2). Among them, the optimal cutoff values of PENK, correlating with the development of AKI, were reported in eight studies, with a mean value of 91.5 pmol/L. The range of sensitivity for identifying AKI lies between 0.55 and 0.90, and the specificity extends from 0.52 to 1.00. The overall sensitivity of PENK for forecasting the occurrence of AKI, as shown in the forest plot, is 0.69 (95% CI 0.62–0.75), while the combined specificity is 0.76 (95% CI 0.68–0.82) (Fig. 1), indicating that PENK has a moderate ability to correctly identify AKI cases and a relatively good ability to accurately identify non-AKI cases. Significant heterogeneity was observed in terms of sensitivity (*I*^2^ = 70.42%, *p* < 0.001) and specificity (*I*^2^ = 93.94%, *p* < 0.001). In regard to the optimal threshold, we employed the different random intercepts and common random slope model to achieve the smallest REML criterion. The determined optimal cutoff value was 57.3 pmol/L.

**Fig. 2:**
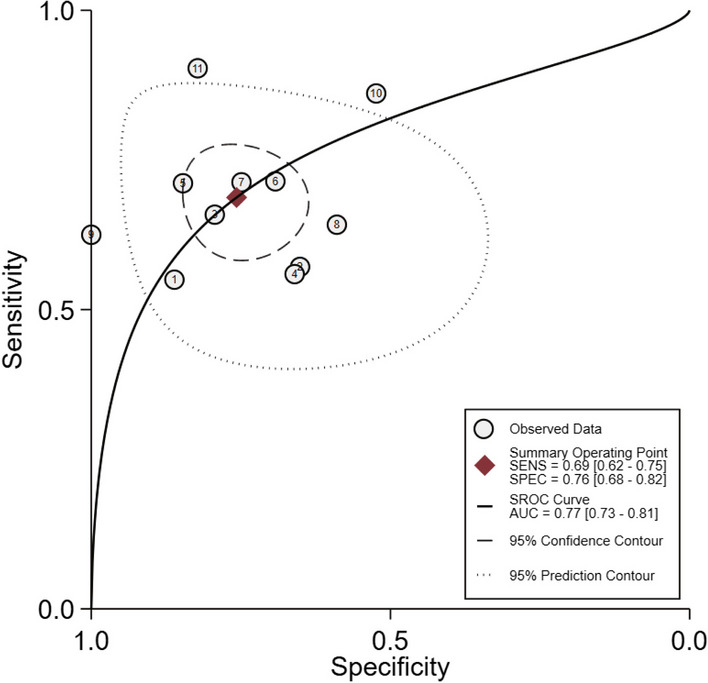
Summary receiver operating characteristic plot of PENK for the early detection of AKI. AKI, Acute kidney injury; PENK, proenkephalin A 119-159

**Fig. 3:**
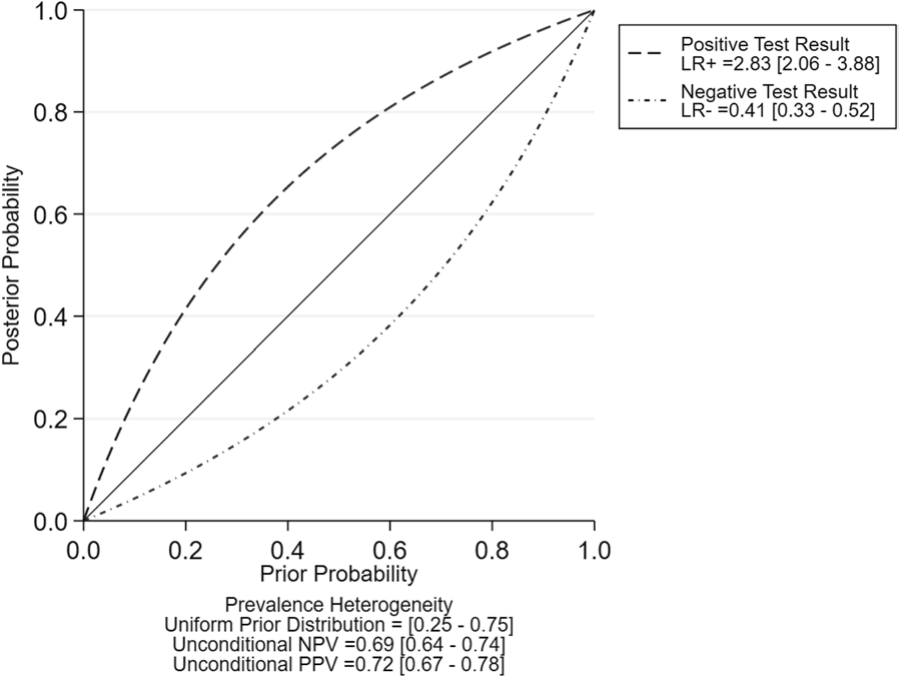
The positive and negative likelihood ratios of PENK diagnostic accuracy for AKI. AKI, Acute kidney injury; LR, likelihood ratio; NPV, negative predictive value; PENK, proenkephalin A 119-159; PPV, positive predictive value

The SROC curve, which illustrates the overall ability of PENK for the early diagnosis of AKI, shows an area under curve (AUC) of 0.77 (95% CI, 0.73–0.81). This suggests that PENK has a moderately accurate discriminatory ability in early detection of AKI (Fig. 2). The positive LR was calculated as 2.83 (95% CI 2.06–3.88), indicating that a positive PENK result increases the likelihood of AKI by approximately threefold. Conversely, the negative LR was found to be 0.41 (95% CI 0.33–0.52) (Fig. 3). Fagan nomograms were utilized to illustrate the effect of positive and negative results on the post-test probability of AKI development. By assuming a pre-test probability of AKI of 25%, based on the observed AKI incidence of 23.4% in this study, the Fagan nomogram demonstrates that when the PENK result is above the cutoff value, the post-test probability of AKI increases to 49%. Conversely, when the PENK result is below the cutoff value, the post-test probability of AKI decreases to 12% (Additional file 1: Fig. S2a). If the pre-test probability for AKI is set at 75%, the post-test probability of AKI increases to 89% when the PENK value is above the cutoff, whereas it decreases to 55% when the PENK value is below the cutoff (Additional file 1: Fig. S2b).

### Patient characteristics and the effect on PENK's diagnostic accuracy for AKI

To explore the potential sources of the heterogeneity in PENK's diagnostic accuracy for AKI, we carried out subgroup analysis and meta-regression. Subgroup analysis was conducted encompassing various variables such as patient characteristics (age, gender, prevalence of HTN, DM and CKD), clinical settings (cardiac events versus non-cardiac events; sepsis versus non-sepsis; surgery versus medical/mixed), AKI severity, AKI definition (KDIGO criteria versus non-KDIGO criteria), follow-up duration and study size (Additional file 1: Table 1). The results derived from the thorough subgroup analysis demonstrate the robust performance of PENK across different patient groups, with the notable exception of those in the large size group.

**Fig. 4:**
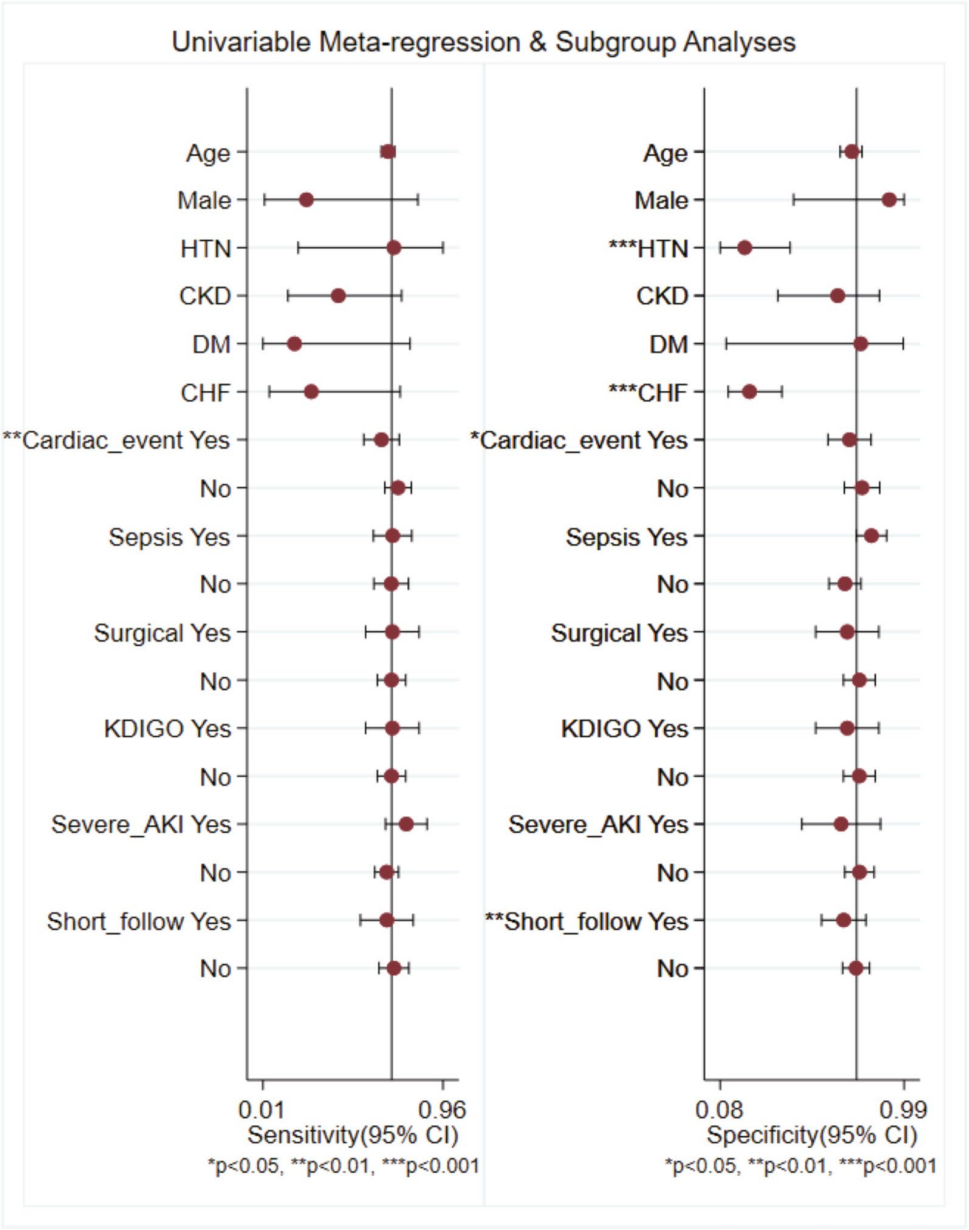
Univariable meta-regression and subgroup analysis for sensitivity and specificity of PENK for the early detection of AKI. AKI, Acute kidney injury; PENK, proenkephalin A 119-159; HTN, hypertension; DM, diabetes mellitus; CHF, congestive heart failure; CI, confidence interval; CRS, cardiorenal syndrome; KDIGO, Kidney Disease: Improving Global Outcomes

Our findings revealed that PENK's overall diagnostic ability for AKI was higher in smaller studies (AUC: 0.81, 95% CI 0.78–0.85) compared to larger counterparts (AUC: 0.71, 95% CI 0.65–0.75). The outcomes stemming from the meta-regression analysis indicate that the coexistence of HTN and CHF appears to reduce in the specificity of PENK's diagnostic capacity concerning the onset of AKI (Fig. 4).

### Trial sequential analysis and the performance of PENK

**Fig. 5:**
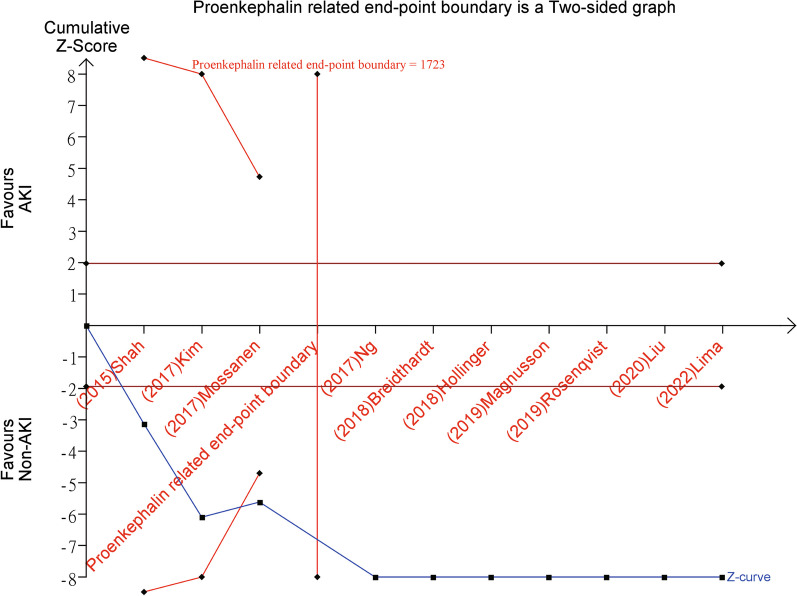
Trial sequential analysis for the efficacy of PENK in early diagnosis of AKI. A penalized test was conducted on the trial sequential analysis (TSA) outcomes, with a predetermined significance level of α = 5% to control for type-I error, a power of 90% to ensure sufficient statistical power, and a two-sided test for the type of bounds. Notably, the penalized Z-curve surpassed the conventional threshold of Z = 1.96, providing additional evidence to support the exclusion of acute kidney injury (AKI) based on the negative levels of proenkephalin A 119-159 (PENK). This reinforces the notion that PENK is an effective marker for ruling out the presence of AKI

The cumulative Z-curve, as analyzed through trial sequential analysis (TSA), demonstrated that the required information size of 1723 patients was exceeded. Moreover, the penalized Z-curve exceeded the conventional threshold value of Z = 1.96, offering additional substantiation for the exclusion of AKI based on negative PENK levels. These findings strongly support the notion that PENK is an effective biomarker for ruling out the presence of AKI and that low levels of PENK provide robust evidence for excluding AKI (Fig. 5).

### Comparisons of diagnostic accuracy between PENK and NGAL

Three studies concurrently evaluated the diagnostic performance of NGAL for AKI [31, 35, 37].

Among these, two studies offered sufficient data to compute the diagnostic odds ratio (DOR) [31, 37]. As illustrated in Additional file 1: Fig. S3, the diagnostic accuracy of PENK for AKI was not inferior to NGAL (*p* = 0.62, test for between group differences (random-effects model)).

### Publication bias

We created Deek’s funnel plot to assess the potential for publication bias, and these plots exhibited largely symmetrical patterns. This finding indicates that the likelihood of publication bias in this meta-analysis is non-significant (*p* = 0.28) (Additional file 1: Fig. S4).

### Assessment of evidence quality and summary of findings

According to the GRADE (Grading of Recommendations, Assessment, Development and Evaluation) framework, the strength of evidence regarding the diagnostic accuracy of PENK for AKI was assessed. The level of certainty surrounding the primary outcome was deemed to be low, primarily because the studies we included were of an observational kind. Despite minimal risk associated with bias, indirectness, imprecision and publication bias, the reliability of the evidence was reduced owing to inconsistencies found within the results of the studies we analyzed (see. Additional file 1).

## Discussion

To the best of our knowledge, this is the first study to examine a meta-analysis on the diagnostic precision of PENK in anticipating incident AKI. We included 11 studies with a total of 3,969 patients and 23.4% patients developed AKI. The meta-analysis revealed PENK’s significant overall accuracy for the early diagnosis of AKI and determined an optimal cutoff point of 57.3 pmol/L. Positive and negative LRs were 2.83 and 0.41, respectively, further reinforcing the reliability and precision of low PENK as a biomarker in “ruling out” AKI (Fig. 3). In considering PENK as a renal function marker, it is plausible that the observed correlation between lower PENK levels and reduced risk of AKI may be partly attributed to the absence of pre-existing CKD in these patients. However, this might not fully explain the observed risk reduction. Hollinger et al. conducted a subset analysis demonstrating that even in patients with low serum creatinine levels at admission, elevated PENK levels remained significantly associated with subsequent AKI [33]. This suggests that the link between PENK levels and AKI risk extends beyond baseline CKD status. Additionally, our findings indicate that PENK's diagnostic performance for AKI is comparable to that of the emerging biomarker NGAL. This result supplements the recent recommendations on AKI biomarkers from the ADQI group by introducing a fresh perspective—indicating that PENK could indeed serve as a robust biomarker for the early detection of AKI [38]. The correlation between AKI and PENK likely stems from shared cellular mechanisms, such as inflammation, that trigger AKI and the release of PENK into the bloodstream [11, 39]. Various mechanisms, such as the effects of toxins, ischemia/reperfusion, activation of neurohormones and inflammation, have all been identified as causing AKI [40, 41]. These results support the use of PENK as a reliable biomarker for AKI as it can reflect reduced filtration and reabsorption in the injured kidney and it can denote upregulation in response to kidney damage [15, 16, 29–37].

### PENK correlating with AKI

Our analyses show that patients with lower PENK levels have a substantially reduced risk of developing AKI. Such negative predictive power provides invaluable insight to clinicians in their decision-making process. The 23rd Acute Disease Quality Initiative (ADQI) consensus group advocates incorporating biomarkers as complementary tools alongside traditional methods for AKI risk stratification, cause identification, severity assessment and prediction of recovery [9]. However, patients with heart failure and HTN can result in a higher false-positive rate when using PENK as an early indicator of AKI. The biological plausibility of this observation stems from the endogenous opioid system in the regulation of cardiovascular function and fluid homeostasis. Notably, elevated levels of PENK have been documented in patients with heart failure and are considered a protective mechanism to counter-regulate the sympathetic nervous system overdrive in the early stages of heart failure [42, 43]. Consequently, this adaptive response may have implications for the performance of PENK as an early indicator for AKI, leading to a higher rate of false-positive results in the presence of heart failure and HTN. Given that AKI is a common among hospitalized patients suffering from HTN or heart failure [40, 44–47], it is warranted to search for more suitable biomarkers in these subgroups. The observed diminished diagnostic performance of PENK for AKI in larger studies merits attention, particularly as these studies included a higher proportion of patients with underlying heart failure. This factor complicates the task of determining whether the variance in diagnostic accuracy is primarily due to inherent small-study effects or is influenced by the elevated prevalence of heart failure in the larger study cohorts [20].

Incorporating PENK into patient care could potentially facilitate the identification of patients at high risk for AKI, who might benefit from more intensive surveillance and personalized prevention efforts. Such strategies may include optimizing fluid status, the judicious use of nephrotoxic agents and prophylactic intravenous hydration prior to contrast media exposure. Further prospective studies are essential to ascertain if PENK-guided interventions truly enhance patient outcomes. Additionally, a deeper understanding of the interactions between the endogenous opioid system, cardiovascular function and PENK's diagnostic performance in AKI is warranted to better inform clinical decision making.

### Limitation

While our study delivers promising outcomes, it is important to acknowledge several limitations. Firstly, the meta-analysis was hampered by moderate sample sizes across most studies, leading to significant heterogeneity. Although no noticeable publication bias was found, the limited number of studies precluded extensive subgroup analysis. Secondly, a variety of PENK analysis methods were employed in these studies, with nine studies using immunoluminometric assays, one study using enzyme-linked immunosorbent assay and one study lacking information on the specific assay used, and thus, determining an optimal PENK cutoff value may prove challenging. Thirdly, it is known that PENK levels are influenced by the glomerular filtration rate (GFR); however, only a few studies addressed this issue and had conflicting results. Two studies concluded that PENK at admission was an independent indicator of AKI, even when accounting for factors such as age, gender, medical history and estimated GFR [33, 37]. Nonetheless, Rosenqvist et al. observed a reduced diagnostic capacity of PENK for AKI when further considering estimated GFR [15]. This discrepancy calls for more research incorporating adjustments for baseline renal function to elucidate the true prognostic value of PENK. Fourthly, evidence from two studies suggested that changes in PENK levels over time may serve as more reliable indicator for AKI development compared to single baseline measurements [16, 29]. Due to the limited number of studies, we were unable to perform an analysis to determine the optimal timing and thresholds for these dynamic changes that would possible enhance the diagnostic accuracy. Furthermore, the small sample sizes in the majority of the included studies could potentially lead to an overestimation of effects and introduce bias due to sampling error [20, 48]. Finally, our meta-analysis exhibited heterogeneity in both pooled sensitivity and specificity, likely due to variations in study design, PENK measurement timing and the method of PENK analysis used. Despite these limitations, our study's conclusions are derived from a variety of studies with differing designs and clinical contexts. Future research should explore how the specific etiology of AKI and its severity affect PENK's diagnostic accuracy. These considerations could be integrated into upcoming randomized controlled trials, aiding in determining optimal cutoff values for various clinical settings, thereby improving the timely diagnosis and management of incident AKI. Additionally, further exploration of the underlying AKI mechanisms might enhance diagnostic performance and timely treatment, potentially reducing the high mortality rate among AKI patients.

## Conclusion

This article synthesizes the findings of a systematic review that suggest PENK as a potential biomarker for incident AKI with high positive and negative LRs. Furthermore, we established a distinct cutoff value for PENK, which enhances its utility in excluding the possibility of AKI. Notably, we determined that its diagnostic accuracy could be comparable to that of NGAL. Although the meta-analysis demonstrates robust overall accuracy, the discrepancies and limitations intrinsic to the included studies, along with the suboptimal diagnostic performance in patients with HTN or heart failure, highlight the necessity for additional clinical trials and real-world studies to validate the utility of PENK as a biomarker for anticipating AKI onset.

### Supplementary Information

**Additional file 1**. Supplementary appendix.

## Data Availability

The datasets used and/or analyzed during the current study are available from the corresponding author on reasonable request.

## Abbreviations

ADQI: Acute Disease Quality Initiative
AKI: Acute kidney injury
AKIN: Acute Kidney Injury Network
AUC: Area under curve
CENTRAL: Cochrane Central Register of Controlled Trials
CHF: Congestive heart failure
CI: Confidence interval
CKD: Chronic kidney disease
CoE: Confidence of evidence
CRS: Cardiorenal syndrome
DM: Diabetes mellitus
DOR: Diagnostic odds ratio
ELISA: Enzyme-linked immunosorbent assay
ESRD: End-stage renal disease
GRADE: Grading of Recommendations, Assessment, Development and Evaluations
GFR: Glomerular filtration rate
HTN: Hypertension
ILMA: Immunoluminometric assay
KDIGO: Kidney Disease: Improving Global Outcomes
LR: Likelihood ratio
N/A: Not applicable
NGAL: Neutrophil gelatinase-associated lipocalin
NPV: Negative predictive value
PENK: Proenkephalin A 119-159
pre-op: Preoperative
post-op: Postoperative
PPV: Positive predictive value
PRISMA: Preferred Reporting Items of Systematic Reviews and Meta-Analyses
QUADAS-2: Quality Assessment of Diagnostic Accuracy Studies-2
RIFLE: Risk, Injury, Failure, Loss, ESRD
RCT: Randomized controlled trial
REML: Restricted maximum likelihood
ROC: Receiver operating characteristic
SCr: Serum creatinine
SROC: Summary receiver operating characteristic
TSA: Trial sequential analysis
