# Supplementary material for: Proenkephalin as a biomarker correlates with acute kidney injury: a systematic review with meta-analysis and trial sequential analysis
Source: Crit Care. 2023 Dec 7;27:481. doi: 10.1186/s13054-023-04747-5 (PMC10702091; doi:10.1186/s13054-023-04747-5)
Supplement: Supplementary file 1 — Additional file 1. Supplementary appendix. [file 13054_2023_4747_MOESM1_ESM.docx]

**Supplementary appendix**

**This supplementary appendix provides:**

1. **Search equation via PubMed, EMBASE, MEDLINE, and Cochrane library**
2. **Flowchart of the updated literature search performed on June 26, 2023**
3. **Quality assessment of the included studies.** A detailed assessment of the quality of the included studies was conducted using the QUADAS-2 (Quality Assessment of Diagnostic Accuracy Studies) tool.
4. **PRISMA checklist**. The PRISMA (Preferred Reporting Items for Systematic Reviews and Meta-Analyses) checklist provides a comprehensive overview of the adherence to reporting guidelines in this meta-analysis.
5. **Supplemental table**
6. **Other supplemental Figures.**
7. **Summary of contextual factor data**
8. **PROSPERO protocol registration.** The PROSPERO (International Prospective Register of Systematic Reviews) protocol registration number is provided, ensuring transparency and accessibility of the study protocol.
9. **The GRADE results.** The GRADE (Grading of Recommendations, Assessment, Development, and Evaluation) results outline the assessment of the overall quality of evidence and the level of certainty in the findings.
10. **Search equation via PubMed, EMBASE, MEDLINE, and Cochrane library**

Search strategies for the different databases ran on **June 26, 2023**

**PubMed (50)**

("acute kidney injury"[MeSH Terms] OR ("acute"[All Fields] AND "kidney"[All Fields] AND "injury"[All Fields]) OR "acute kidney injury"[All Fields] OR "acute kidney injury"[MeSH Terms] OR ("acute kidney injury"[MeSH Terms] OR ("acute"[All Fields] AND "kidney"[All Fields] AND "injury"[All Fields]) OR "acute kidney injury"[All Fields] OR ("acute"[All Fields] AND "renal"[All Fields] AND "failure"[All Fields]) OR "acute renal failure"[All Fields]) OR "acute kidney injury"[MeSH Terms] OR (("acute"[All Fields] OR "acutely"[All Fields] OR "acutes"[All Fields]) AND ("kidney"[MeSH Terms] OR "kidney"[All Fields] OR "kidneys"[All Fields] OR "kidney s"[All Fields]) AND ("impair"[All Fields] OR "impaired"[All Fields] OR "impairement"[All Fields] OR "impairements"[All Fields] OR "impairing"[All Fields] OR "impairment"[All Fields] OR "impairments"[All Fields] OR "impairs"[All Fields])) OR ("acute kidney injury"[MeSH Terms] OR ("acute"[All Fields] AND "kidney"[All Fields] AND "injury"[All Fields]) OR "acute kidney injury"[All Fields] OR ("acute"[All Fields] AND "kidney"[All Fields] AND "insufficiency"[All Fields]) OR "acute kidney insufficiency"[All Fields]) OR "acute kidney injury"[MeSH Terms] OR "AKI"[All Fields]) AND ("Proenkephalin"[Supplementary Concept] OR "Proenkephalin"[All Fields] OR "proenkephalins"[All Fields] OR ("Proenkephalin"[Supplementary Concept] OR "Proenkephalin"[All Fields] OR "proenkephalin a"[All Fields]) OR (("Proenkephalin"[Supplementary Concept] OR "Proenkephalin"[All Fields] OR "proenkephalin a"[All Fields]) AND "119-159"[All Fields]) OR "PenKid"[All Fields] OR "PENK"[All Fields])

**Embase (72)**

#1. 'acute kidney failure'/exp

#2. 'acute kidney injury'

#3. 'acute kidney impairment'

#4. 'acute kidney insufficiency'

#5. #1 OR #2 OR #3 OR #4

#6. 'proenkephalin'/exp

#7. 'proenkephalin a'

#8. 'proenkephalin a 119-159'

#9. 'penkid'

#10. 'penk'

#11. #6 OR #7 OR #8 OR #9 OR #10

#12. #5 AND #11

**Medline (EBSCO) Search Query (37)**

((((("Acute kidney injury"[Mesh]) OR (acute renal failure)) OR (acute kidney impairment)) OR (acute kidney insufficiency)) OR (AKI)) AND (((((Proenkephalin) OR (Proenkephalin A)) OR (Proenkephalin A 119-159)) OR (PenKid)) OR (PENK))

**Cochrane library (8)**

#1 MeSH descriptor: [Acute Kidney Injury] explode all trees

#2 acute renal failure

#3 acute kidney impairment

#4 acute kidney insufficiency

#5 AKI

#6 #1 or #2 or #3 or #4 or #5

#7 Proenkephalin

#8 Proenkephalin A

#9 "Proenkephalin A 119-159"

#10 PenKid

#11 PENK

#12 #7 or #8 or #9 or #10 or #11

#13 #6 and #12

1. **Flowchart of study selection for meta-analysis.**

**
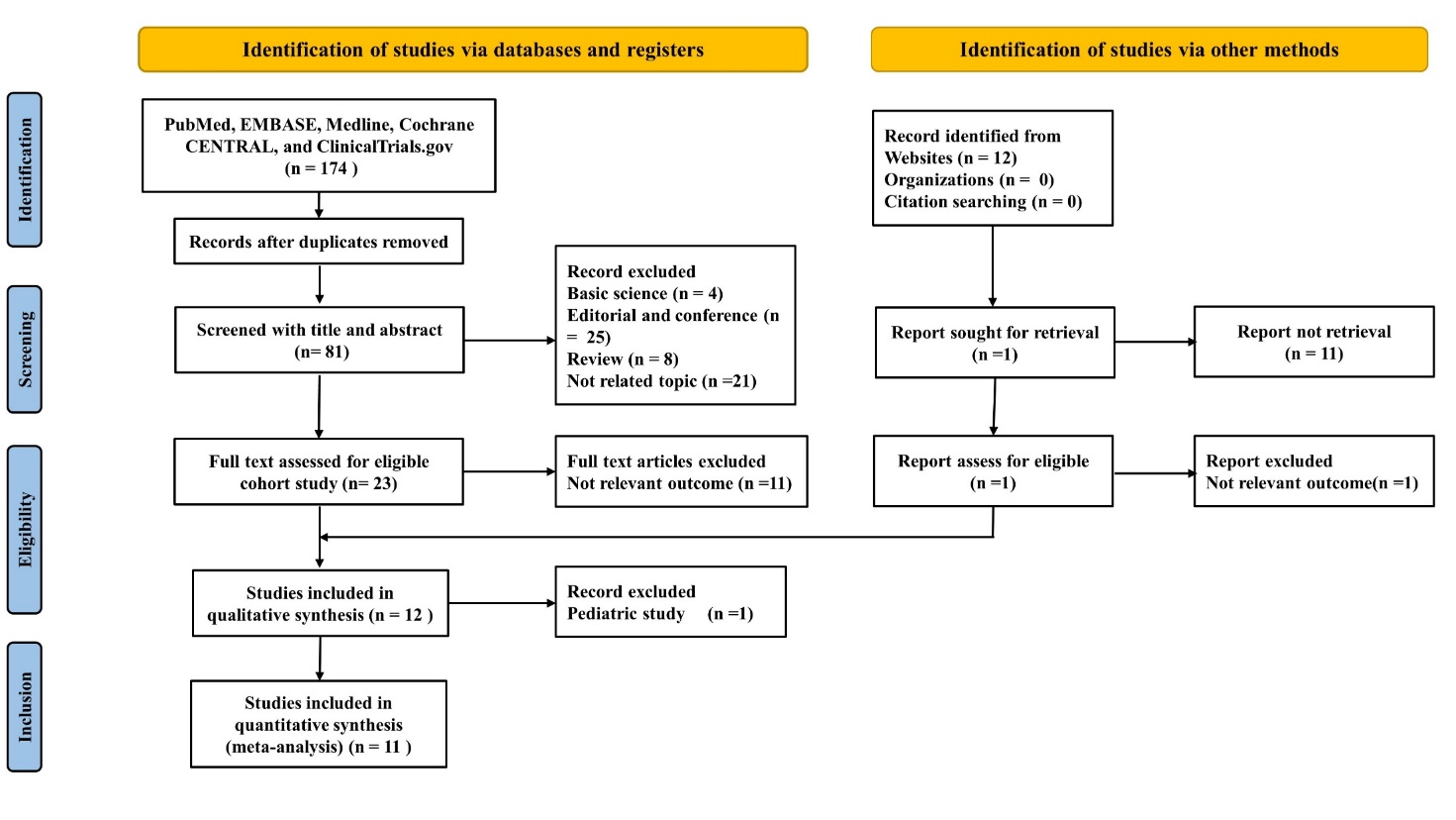
**

1. **Quality assessment of the included studies**

**Supplemental Figure 1**: **Risk of bias and applicability concerns using the QUADAS-2 tool for each included study based on the complete literature-based analysis**

**
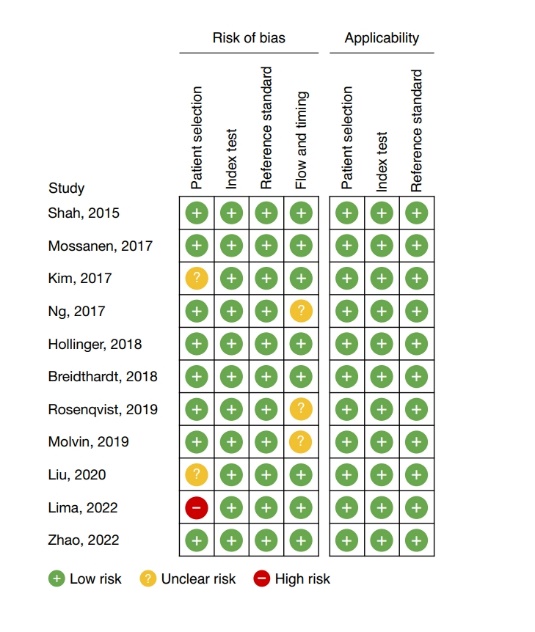
**

1. **PRISMA checklist**

1. **Supplemental table**

**Supplementary Table 1. Subgroup analysis**

| **Subgroup factors** | **Subgroup criteria** | **No. of Studies** | **No. of Patients** | **AKI (%)** | **Sensitivity (95%CI)** | ***I^2^*(%)** | **Specificity (95% CI)** | ***I^2^* (%)** | **AUC (95%)** |
| --- | --- | --- | --- | --- | --- | --- | --- | --- | --- |
| Cardiac events | With cardiac events | 5 | 2422 | 375 (15.5) | 0.66 (0.53-0.77) | 79.71 | 0.72 (0.61-0.81) | 94.61 | 0.75 (0.64-0.83) |
|  | Without cardiac events | 6 | 1547 | 544 (35.8) | 0.73 (0.66-0.78) | 5.04 | 0.79 (0.65-0.88) | 82.25 | 0.79 (0.75-0.82) |
| Sepsis | Only sepsis patients | 4 | 1379 | 511 (37.0) | 0.70 (0.66-0.74) | 0.00 | 0.83 (0.71-0.91) | 82.81 | 0.73 (0.68-0.76) |
|  | Not only sepsis patients | 7 | 2590 | 387 (14.9) | 0.70 (0.57-0.80) | 79.71 | 0.70 (0.62-0.77) | 92.09 | 0.76 (0.72-0.79) |
| Patient type | Surgery* | 3 | 256 | 77 (30.1) | N/A | N/A | N/A | N/A | N/A |
|  | Medical/mixed | 8 | 3713 | 852 (22.9) | 0.68 (0.61-0.74) | 72.69 | 0.77 (0.69-0.83) | 95.54 | 0.77 (0.73-0.80) |
| Mean age | Low (≤ 72.7) | 6 | 1126 | 509 (45.2) | 0.73 (0.62-0.82) | 66.48 | 0.78 (0.69-0.85) | 81.05 | 0.82 (0.78-0.85) |
|  | High (> 72.7) | 4 | 2801 | 404 (14.4) | 0.64 (0.55-0.72) | 67.75 | 0.71 (0.61-0.78) | 94.32 | 0.71 (0.69-0.74) |
| Male percentage | Low (≤ 61.5%) | 5 | 1463 | 269 (18.4) | 0.75 (0.65-0.84) | 68.62 | 0.72 (0.62-0.80) | 93.42 | 0.80 (0.75-0.85) |
|  | High (> 61.5%) | 5 | 2467 | 644 (26.1) | 0.64 (0.57-0.71) | 74.55 | 0.77 (0.68-0.84) | 95.85 | 0.74 (0.63-0.83) |
| HTN prevalence | Low (≤ 71%) | 4 | 2367 | 647 (27.3) | 0.68 (0.59-0.76) | 89.54 | 0.80 (0.71-0.87) | 97.99 | 0.80 (0.69-0.87) |
|  | High (> 71%)* | 3 | 748 | 95 (14.7) | N/A | N/A | N/A | N/A | N/A |
| DM prevalence | Low (≤ 33%) | 4 | 2863 | 721 (25.2) | 0.69 (0.59-0.77) | 88.59 | 0.77 (0.69-0.84) | 97.18 | 0.79 (0.75-0.83) |
|  | High (> 33%) | 4 | 840 | 115 (14.0) | 0.60 (0.49-0.70) | 0.00 | 0.74 (0.0.61-0.84) | 94.20 | 0.64 (0.54-0.74) |
| CKD prevalence | Low (≤ 30%) | 6 | 2020 | 593 (29.3) | 0.69 (0.64-0.74) | 58.11 | 0.76 (0.67-0.83) | 94.30 | 0.74 (0.70-0.78) |
|  | High (> 30%)* | 2 | 1683 | 243 (14.4) | N/A | N/A | N/A | N/A | N/A |
| AKI severity | Any stage AKI | 9 | 3324 | 799 (24.0) | 0.66 (0.59-0.72) | 68.68 | 0.77 (0.69-0.84) | 95.19 | 0.75 (0.71-0.78) |
|  | Stage 2 or 3 AKI* | 2 | 645 | 130 (20.2) | N/A | N/A | N/A | N/A | N/A |
| AKI definition | KDIGO | 7 | 2648 | 741 (28.0) | 0.71 (0.60-0.80) | 81.88 | 0.78 (0.66-0.86) | 95.69 | 0.80 (0.71-0.87) |
|  | Not KDIGO | 4 | 1321 | 188 (14.2) | 0.67 (0.60-0.74) | 0.00 | 0.72 (0.62-0.81) | 93.16 | 0.68 (0.64-0.72) |
| Follow-up duration | Shorter (≤ 2 days)* | 3 | 808 | 115 (14.2) | N/A | N/A | N/A | N/A | N/A |
|  | Longer (> 2 days) | 7 | 3119 | 798 (39.0) | 0.70 (0.60-0.79) | 82.12 | 0.75 (0.67-0.82) | 94.34 | 0.80 (0.76-0.83) |
| Study size | Small (≤ 200) | 7 | 697 | 172 (24.7) | 0.72 (0.60-0.82) | 60.82 | 0.78 (0.66-0.87) | 80.93 | 0.81 (0.78-0.85) |
|  | Large (> 200) | 4 | 3272 | 757 (23.1) | 0.64 (0.56-0.71) | 82.12 | 0.72 (0.62-0.81) | 95.92 | 0.71 (0.67-0.75) |

**Abbreviations:** AKI, acute kidney injury; AUC, area under curve; CI, confidence interval; CKD, chronic kidney disease; DM, diabetes mellitus; HTN, hypertension; KDIGO, Kidney Disease: Improving Global Outcomes; N/A, not applicable

* Due to the insufficient number of studies, it was not feasible to generate pooled results from a meta-analysis.

1. **Other Supplemental Figures**

**Supplemental Figure 2. Bayesian analysis of the clinical applicability of PENK for the early detection of AKI.**

1. **With a pretest probability of 25% for AKI**


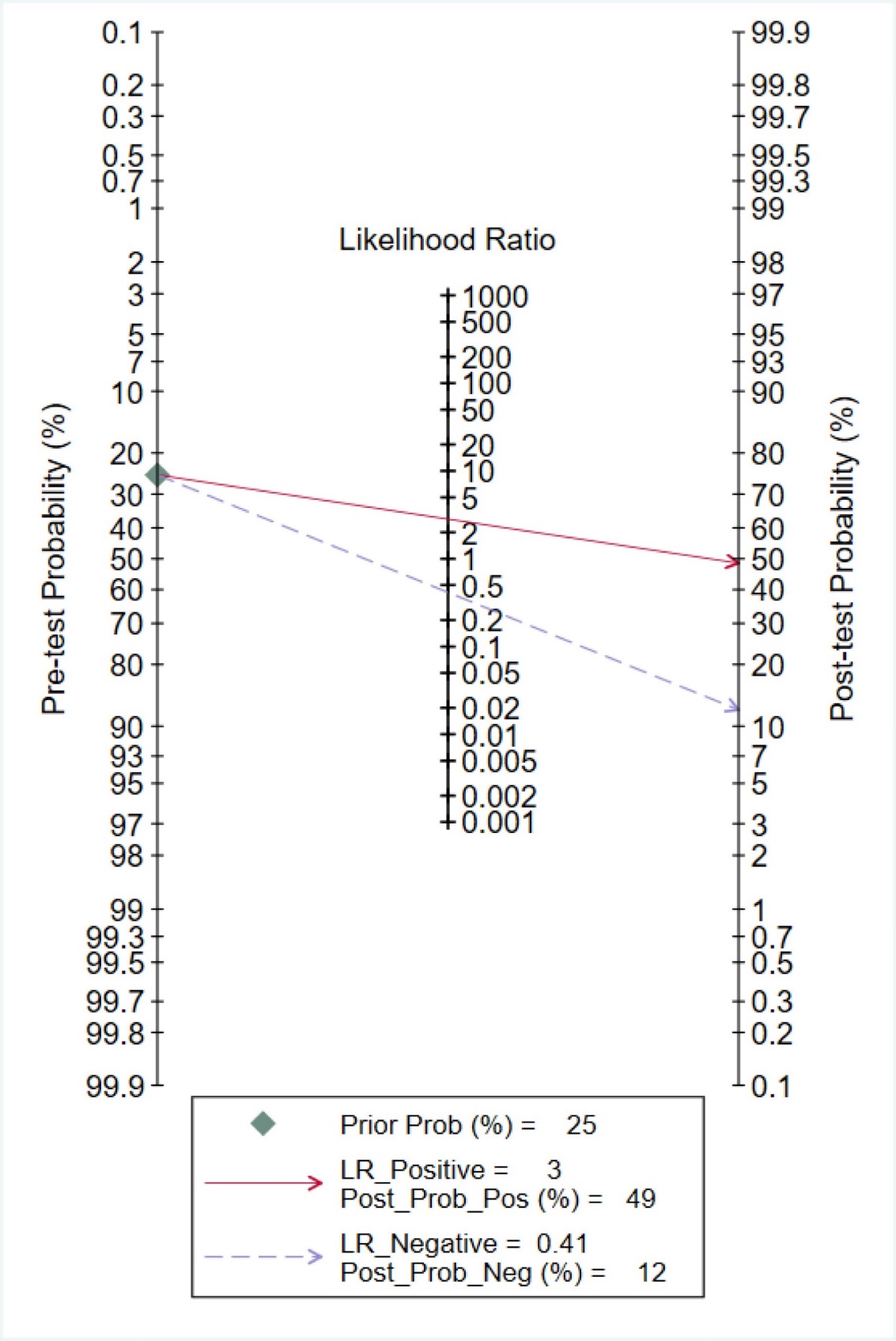


1. **With a pretest probability of 75% for AKI**

**
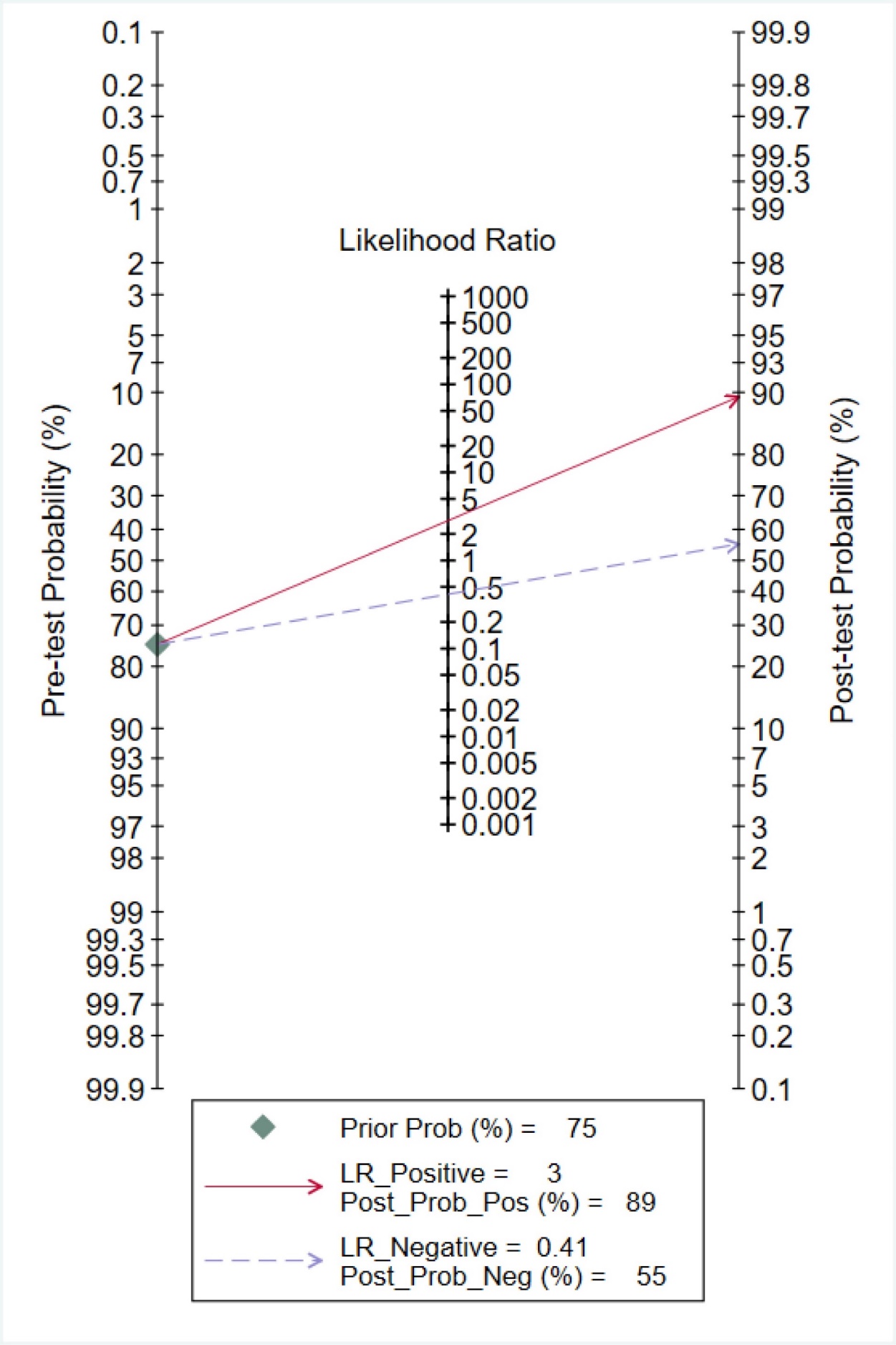
**

**Abbreviations:** AKI, acute kidney injury; LR, likelihood ratio; PENK, proenkephalin A 119-159


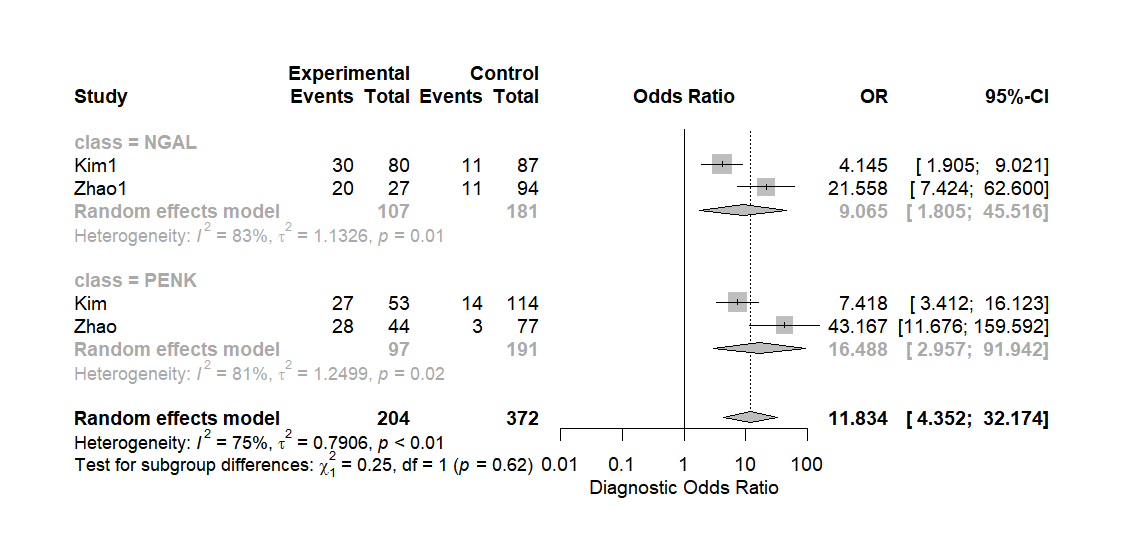
**Supplemental Figure 3: The comparison of the diagnostic odds ratio between PENK and NGAL for AKI**

**Abbreviations:** AKI, acute kidney injury; DOR, diagnostic odds ratio; NGAL, neutrophil gelatinase-associated lipocalin; PENK, proenkephalin A 119-159

**Supplemental Figure 4: Deeks’ funnel plot for the assessment of potential publication bias**


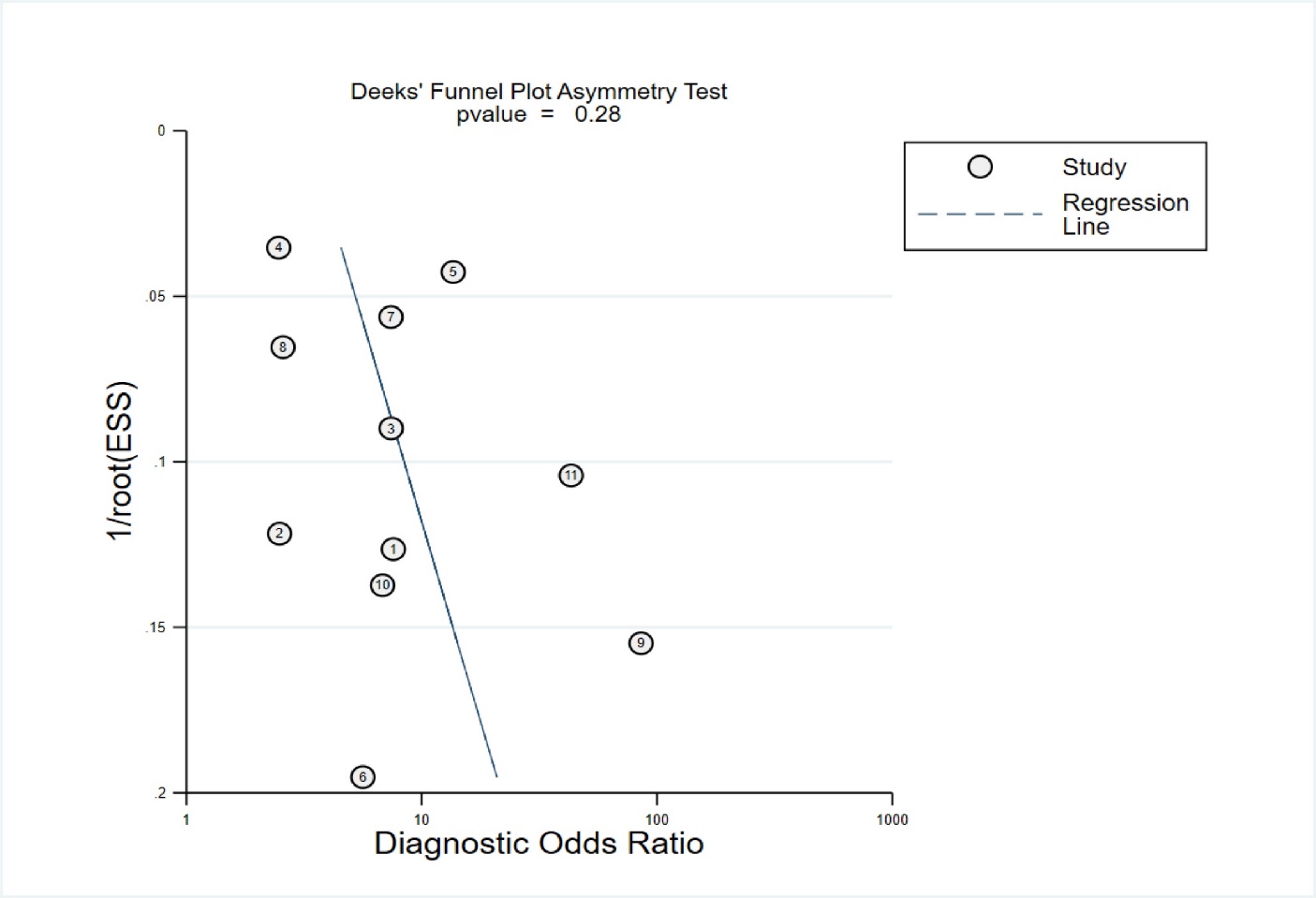


**Supplemental Figure 5: Graphic abstract**

**
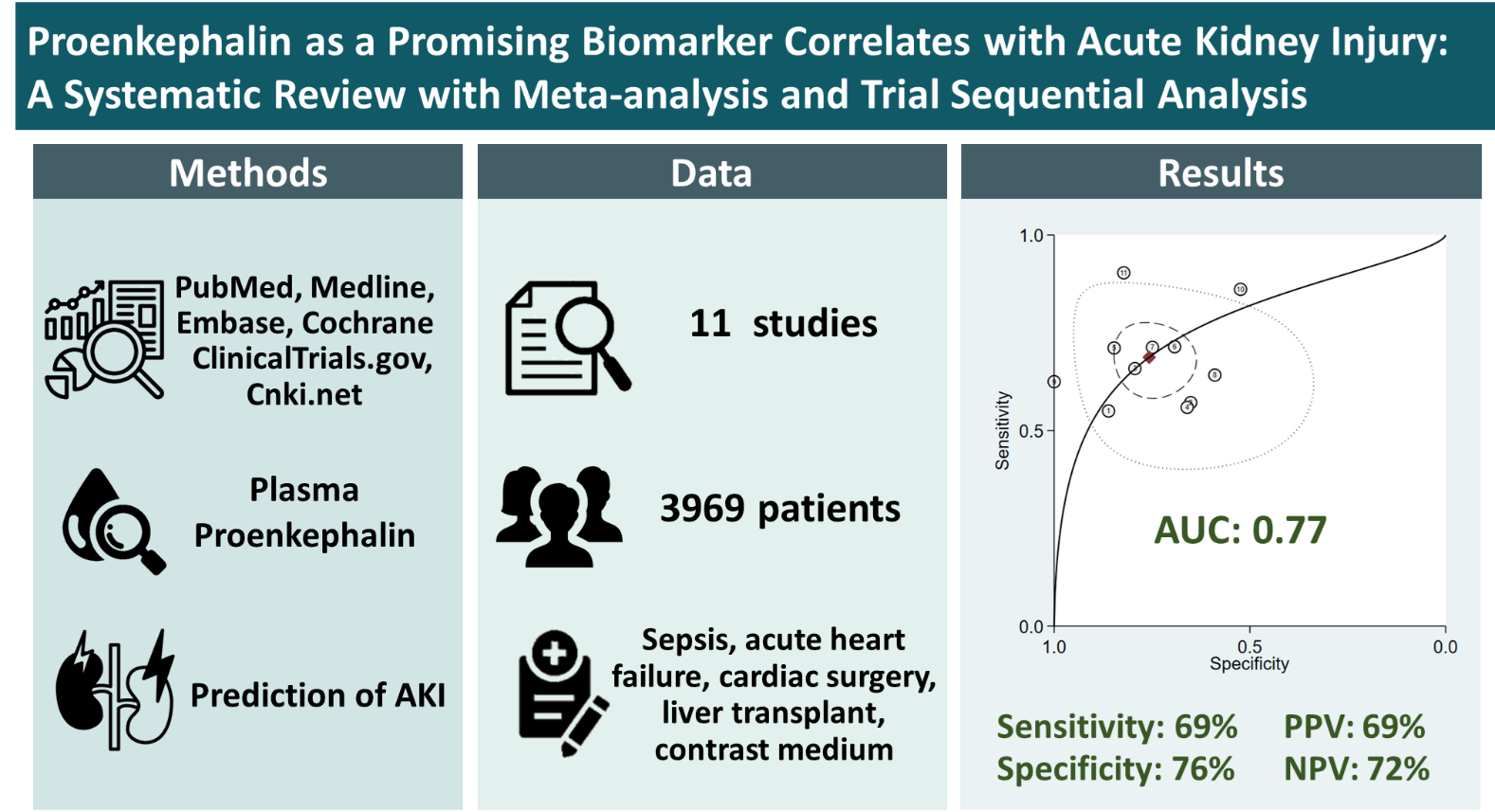
**

**Abbreviations:** AKI, acute kidney injury; PPV, positive predictive value; NPV, negative predictive value

1. **Summary of contextual factor data**

The present meta-analysis incorporated a total of 11 studies, comprising 3,969 patients, to comprehensively evaluate the predictive performance of proenkephalin A 119-159 (PENK) for acute kidney injury (AKI). Notably, each study contributed unique insights, shedding light on the association between PENK levels and AKI across various clinical scenarios.

**Shah et al. (2015)** investigated 92 patients undergoing cardiac surgery, revealing a significant association between pre-operative PENK levels and post-operative AKI. Although the optimal cutoff value was not provided, sensitivity and specificity data were extracted from the receiver operating characteristic (ROC) curve using WebPlotDigitizer 4.4, yielding a sensitivity of 55% and a specificity of 86%. The study demonstrated an area under curve (AUC) of 0.683.

In the study by **Mossanen et al. (2017)**, which involved 107 patients undergoing elective cardiac surgery, the optimal cutoff value for pre-operative PENK was determined to be 93.2 pmol/L. This cutoff value achieved a sensitivity of 59% and specificity of 65% for predicting post-operative AKI. The AUC was reported as 0.651.

**Kim et al. (2017)** enrolled 167 septic patients and compared the predictive value of plasma PENK with plasma neutrophil gelatinase-associated lipocalin (NGAL) for AKI. Their findings indicated that PENK outperformed NGAL, exhibiting an AUC of 0.725 compared to 0.675 for NGAL. The optimal cutoff value for PENK in predicting AKI was determined to be 154.5 pmol/L, with a sensitivity of 65.9% and a specificity of 79.4%.

**Ng et al. (2017)** enrolled 1,908 patients with acute heart failure and examined the prognostic value of PENK. While the primary outcome was all-cause 1-year mortality, the study also evaluated the prediction of AKI as a secondary endpoint. Initially, the article did not provide specific details on the AUC, cutoff value, sensitivity, or specificity of PENK for AKI prediction. However, upon contacting the first author and redefining the outcome to focus on AKI based on Kidney Disease: Improving Global Outcomes (KDIGO) criteria, a reanalysis involving 1,572 patients yielded an AUC of 0.642, a sensitivity of 56%, and a specificity of 66% using a threshold value of 116.7 pmol/L for AKI prediction.

**Hollinger et al. (2018)** included 582 septic patients and determined the optimal cutoff value for PENK to be 84.2 pmol/L in predicting persistent AKI by day 7. At this threshold, PENK demonstrated a sensitivity of 71.1%, specificity of 84.7%, and an AUC of 0.854.

**Breidthardt et al. (2018)** examined 111 patients with chronic kidney disease undergoing contrast procedures. While baseline PENK exhibited poor predictive performance for contrast-induced AKI (AUC = 0.60, sensitivity = 71%, specificity = 69%), day 1 PENK and delta PENK (change in PENK levels between baseline and day 1) demonstrated predictive value, with AUCs of 0.79 and 0.92, respectively. The article did not provide information regarding the optimal cutoff point for PENK.

**Rosenqvist et al. (2019)** enrolled 588 patients with sepsis, reporting an AUC of 0.758 for PENK in predicting AKI. The optimal cutoff value was not provided in the article, but sensitivity and specificity values were extracted from the ROC curve using WebPlotDigitizer.

**Molvin et al. (2019)** enrolled 530 patients with acute heart failure. PENK was associated with

AKI, with an AUC of 0.65. AKI was defined as an increase in serum creatinine by ≥ 0.3 mg/dL or ≥ 50% higher than the baseline value within a 48-hour period. Specific data regarding the cutoff value of PENK for predicting AKI, as well as the associated sensitivity and specificity, were obtained by contacting the first author. The cutoff value was determined to be 55.3 pmol/L, with a sensitivity of 64% and a specificity of 59%.

**Liu et al. (2020)** enrolled 42 patients with sepsis. PENK showed predictive value for AKI, with an AUC of 0.884. An optimal cutoff value for PENK was identified at 66.97 pmol/L, demonstrating a sensitivity of 100% and a specificity of 91.4% in predicting AKI.

**Lima et al. (2022)** enrolled 57 patients undergoing liver transplantation. Pre-operative PENK levels were identified as predictors of post-operative severe AKI (KDIGO stage 2-3) with an AUC of 0.69. The optimal cutoff value for PENK was determined to be 55.30 pmol/L, with a sensitivity of 86% and a specificity of 52%.

**Zhao et al. (2023)** enrolled 121 patients with acute decompensated heart failure. PENK showed predictive efficiency for type 1 cardiorenal syndrome, achieving an AUC of 0.808.

At an optimal cutoff point of 57.0 pmol/L, PENK had a sensitivity of 90% and a specificity of 82%.

1. **PROSPERO protocol registration**

PROSPERO 2023 CRD42023424693
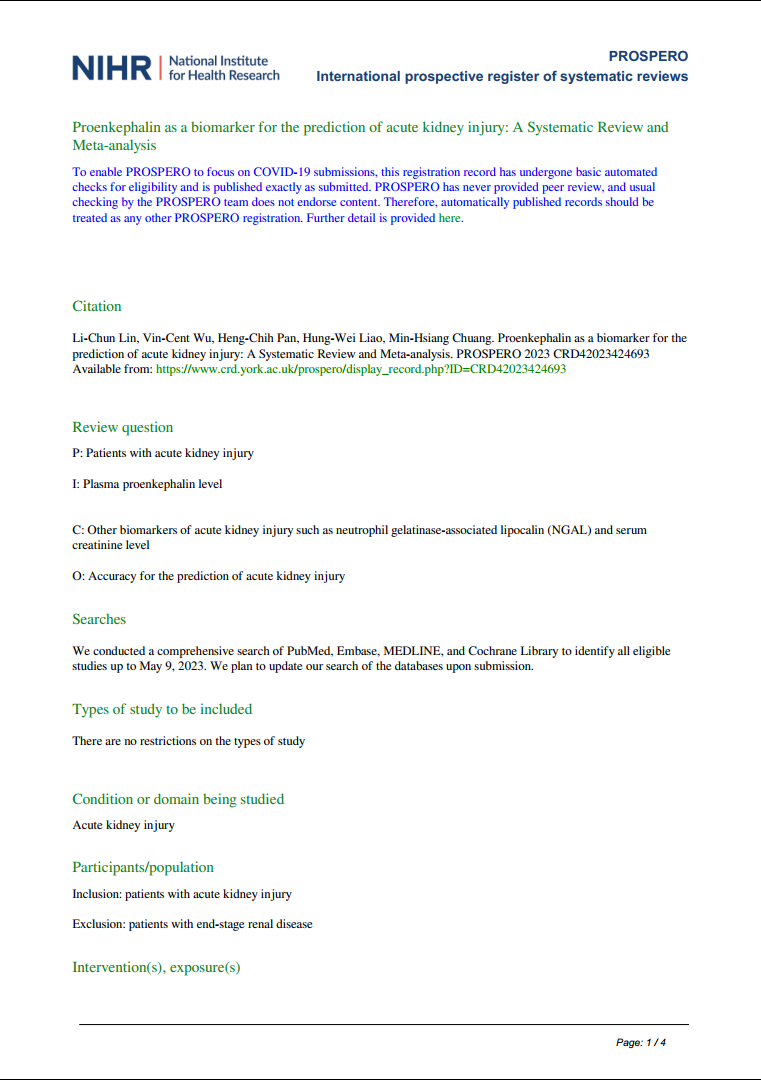


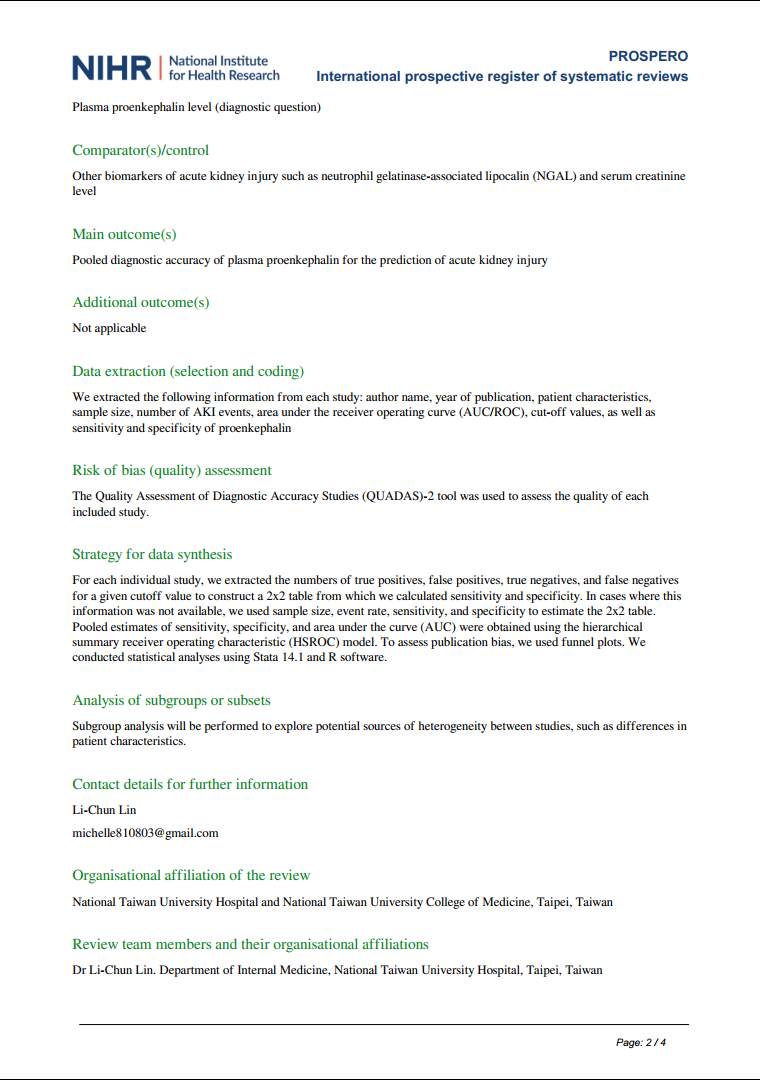


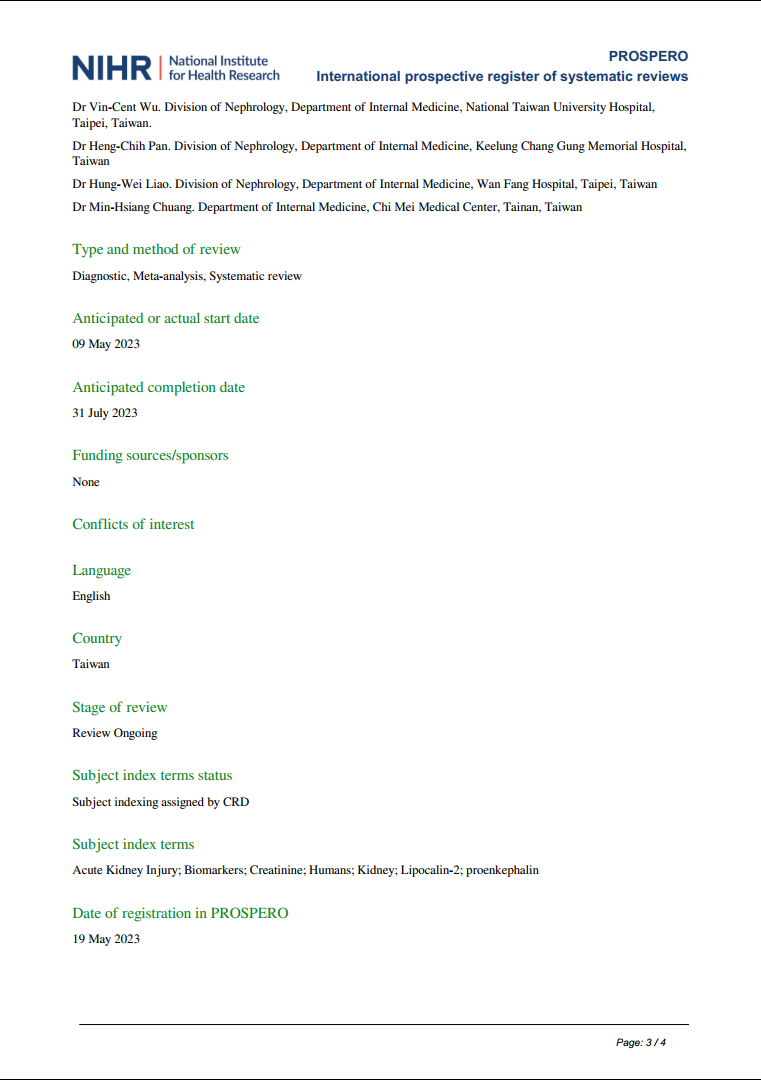


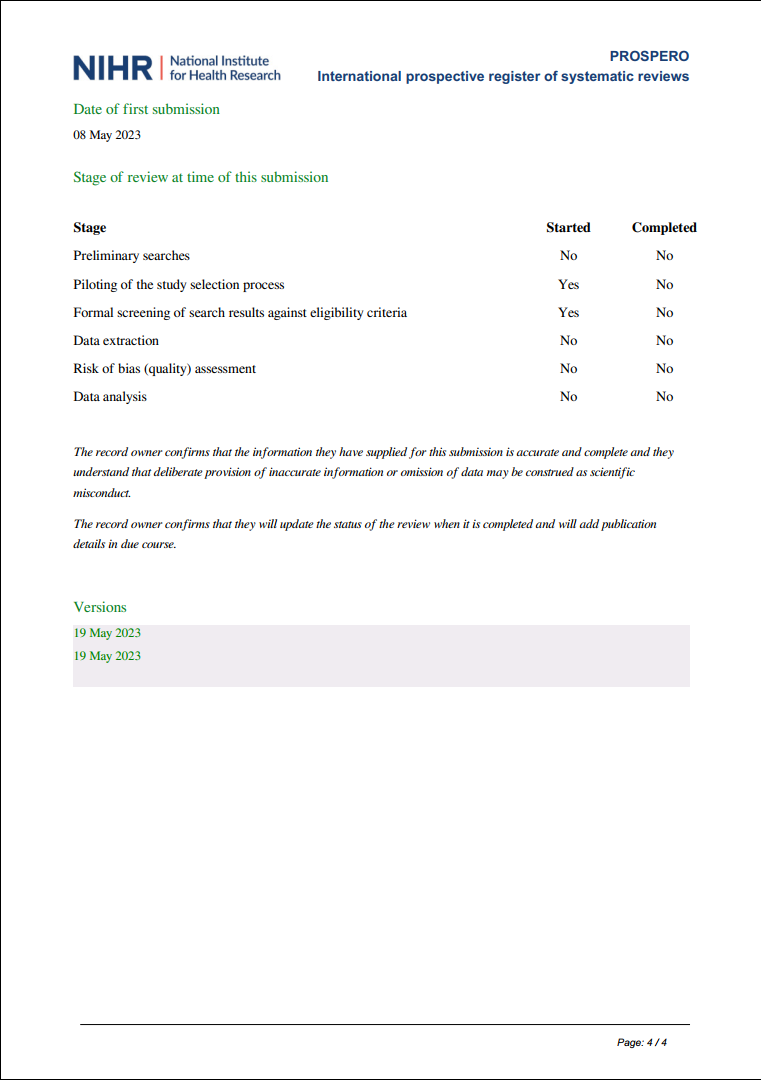


1. **The GRADE assessment**

**Supplemental Table 1. Certainty of evidence assessment with GRADE approach**

| **Question**: Should Proenkephalin A 119-159 be used for the early detection of AKI? | | | | | | | | | | | |
| --- | --- | --- | --- | --- | --- | --- | --- | --- | --- | --- | --- |
| Sensitivity | 0.69 (95% CI: 0.62 to 0.75) | | | | | | | | | | |
| Specificity | 0.76 (95% CI: 0.68 to 0.82) | | | | | | | | | | |
| Outcome | No. of studies (No. of patients) | Study design | Factors that may decrease certainty of evidence | | | | | Effect per 1,000 patients tested | | | Test accuracy CoE |
|  |  |  | Risk of bias | Indirectness | Inconsistency | Imprecision | Publication bias | Pre-test probability of 25% | | Pre-test probability of 75% |  |
| **True positives** (patients with AKI) | 11 studies 929 patients | cross-sectional (cohort type accuracy study) | not serious | not serious | serious^a^ | not serious | none | 173 (155 to 188) | 518 (465 to 563) | | ⨁⨁○○ Low |
| **False negatives** (patients incorrectly classified as not having AKI) |  |  |  |  |  |  |  | 77 (63 to 95) | 232 (188 to 285) | |  |
| **True negatives** (patients without AKI) | 11 studies 3040 patients | cross-sectional (cohort type accuracy study) | not serious | not serious | very serious^a^ | not serious | none | 570 (510 to 615) | 190 (170 to 205) | | ⨁⨁○○  Low |
| **False positives** (patients incorrectly classified as having AKI) |  |  |  |  |  |  |  | 180 (135 to 240) | 60 (45 to 80) | |  |

**Abbreviations:** AKI, acute kidney injury; AKI, acute kidney injury; CoE, confidence of evidence; GRADE, Grading of Recommendations, Assessment, Development and Evaluations

a. High *I^2^* value
